# Graphene Nanogrids FET Immunosensor: Signal to Noise Ratio Enhancement

**DOI:** 10.3390/s16101481

**Published:** 2016-10-08

**Authors:** Jayeeta Basu, Chirasree RoyChaudhuri

**Affiliations:** Electronics and Telecommunication Engineering Department, Indian Institute of Engineering Science and Technology, Howrah 711103, India; pal.joyeeta@gmail.com

**Keywords:** graphene nanosensor, FET, immunosensor, low frequency noise, sensitivity, cross linker, optimization

## Abstract

Recently, a reproducible and scalable chemical method for fabrication of smooth graphene nanogrids has been reported which addresses the challenges of graphene nanoribbons (GNR). These nanogrids have been found to be capable of attomolar detection of biomolecules in field effect transistor (FET) mode. However, for detection of sub-femtomolar concentrations of target molecule in complex mixtures with reasonable accuracy, it is not sufficient to only explore the steady state sensitivities, but is also necessary to investigate the flicker noise which dominates at frequencies below 100 kHz. This low frequency noise is dependent on the exposure time of the graphene layer in the buffer solution and concentration of charged impurities at the surface. In this paper, the functionalization strategy of graphene nanogrids has been optimized with respect to concentration and incubation time of the cross linker for an enhancement in signal to noise ratio (SNR). It has been interestingly observed that as the sensitivity and noise power change at different rates with the functionalization parameters, SNR does not vary monotonically but is maximum corresponding to a particular parameter. The optimized parameter has improved the SNR by 50% which has enabled a detection of 0.05 fM Hep-B virus molecules with a sensitivity of around 30% and a standard deviation within 3%. Further, the SNR enhancement has resulted in improvement of quantification accuracy by five times and selectivity by two orders of magnitude.

## 1. Introduction

Graphene is a planar single sheet of sp^2^-bonded carbon atoms arranged in a honeycomb lattice. Though graphene is intrinsically a zero band gap material, its band gap and carrier concentration can be tailored to a few eV by applying external electric field or by surface adsorption of chemical elements. This feature makes graphene a flexible material for the development of superior electronic devices [1,2,3]. Such tunable and diverse properties of graphene provide vast possibilities in various sensing applications [2,3]. However, in planar graphene, the transconductance to conductance ratio is usually low which deteriorates the performance of the field effect transistor (FET) biosensors [4]. In this aspect, etching nanostructures in bulk graphene can be advantageous since it introduces a size dependent effective energy gap [5].

Nanostructures in graphene are in the form of nanoribbons, fabricated by either lithographic patterning of graphene sheets or by chemical processes. The former technique usually yields rough edges with dangling bonds and defects [4]. Such inactive edges decrease the effective channel width of the nanoribbon FETs and hence are a major disadvantage for achieving low detection limits [5]. This may be primarily attributed to the presence of active chemical oxygen groups required for thiol functionalization near the edges, which if they cannot contribute towards the effective channel conductance, a significant number of immobilized biomolecules become redundant, suppressing the overall sensitivity [6]. Chemically derived GNRs can circumvent this problem since they have ultra-smooth edges [7,8,9], but as they are dispersed in solution, it requires dip coating or spinning to transfer them to silicon oxide substrates, resulting in totally random orientations of the GNRs. This hinders the reliable placement of GNRs to form a continuous network, restricting the detection limit to a few µM in FET biosensors [9]. These challenges of graphene nanostructures have been addressed recently by a reliable and scalable chemical method for fabrication of smooth graphene nanogrids which has resulted in attomolar sensitivity of a FET biosensor [10]. This has been attributed to the combined effects of insignificant line edges, quantum dot-like transport behaviors and improved interaction of the biomolecules within the nanopores. However, for sub-femtomolar detection of target molecules, it is not sufficient to only explore the steady state sensitivities but is also necessary to investigate the flicker noise which dominates the noise spectrum at frequencies below 100 kHz. This low frequency noise in liquid gated condition which is dependent on the concentration of impurities at the surface and exposure time to solution [11,12] is an important parameter for determining the sensor resolution [13,14].

To date, most of the studies on graphene noise have been conducted on mechanically exfoliated single layer and bilayer devices [15,16,17,18,19]. It has been observed that the noise is usually smaller in bilayer structures compared to single layer devices. The noise is dependent on the gate-induced carrier density and hence is expected to vary with the charge concentration at the surface [15]. Further, the noise magnitude has also been reported to depend on the disorder level, thickness of the grain boundaries [16] and also on the substrate on which it has been deposited [18,19]. Tan et al. report that nanoribbons show lower flicker noise levels than planar graphene but the shot noise increases in graphene nanoribbons due to increased scattering in the presence of disordered edges [20,21,22]. Moreover, in bilayer graphene nanoribbons, it has been observed that the noise increases with increasing carrier density [23,24]. Thus it may be envisaged that, tailoring the carrier density in smooth graphene nanogrid structures can adjust the noise levels in the drain current and result in the development of a high performance sensor with an optimized SNR.

This paper makes the first attempt to enhance the SNR in graphene nanosensors operating in FET mode by controlling the functionalization protocol. For this purpose, graphene nanogrids FET immunosensor has been developed following a similar methodology reported earlier [10]. This has been followed by optimizing the incubation time and concentration of glutaraldehyde as the crosslinker. Glutaraldehyde has been widely used as crosslinking agent for immobilizing biomolecules [25,26,27]. In this work, the impact of optimization has been studied by estimating both the steady state sensitivity and noise power spectral density (PSD) from the drain current characteristics for each of the functionalization parameters, followed by calculation of their ratio to obtain SNR, with varying Hep-B virus concentration. Reliability, quantification accuracy and selectivity have been evaluated for the optimized and un-optimized sensors to establish the influence of SNR towards realization of reproducible graphene nanogrid sensor with sub-femtomolar detection limit.

## 2. Materials and Methods

### 2.1. Graphene Nanogrid Fabrication

For fabrication of graphene nanogrid structure, firstly nanoporous silicon oxide (NPSO) substrate has been developed by anodic etching of p-type <100> silicon wafers of 10–20 Ω·cm resistivity in a double pond electrochemical bath for 30 min under a constant current source with an electrolyte mixture of hydrofluoric acid (HF) (48 wt%) and dimethyl sulfoxide (DMSO) in the ratio of 1:9 by volume. The structure has been thermally oxidized using a dry-wet-dry sequence in an oxidation furnace for 1 h at 900 °C to obtain pores with 30 nm diameter (*d*) and 100 nm length (*l*). Secondly, bilayer graphene has been deposited on nanoporous silicon oxide substrate by electrophoretic deposition (EPD) method. For EPD of graphene, interdigited metal electrodes have been fabricated with high temperature silver paste by screen printing method which has been next cured at 750 °C for 1 min. This has been followed by evaporation of gold metal. The prepared colloidal solution of graphene has been pipetted onto the substrate. Then the electrodes have been connected with two probes of function generator and a sinusoidal voltage (peak to peak 19.5 V) has been applied for 60 s. The graphene deposited substrate has been heated at 75 °C for 2 min. This step increases the adhesion between graphene and the substrate. The aforesaid fabrication results in a nanogrid structure with long and narrow graphene strips alternating with series combination of short and narrow strips on planar regions and within pores.

### 2.2. Immobilization Process

**Figure 1:**
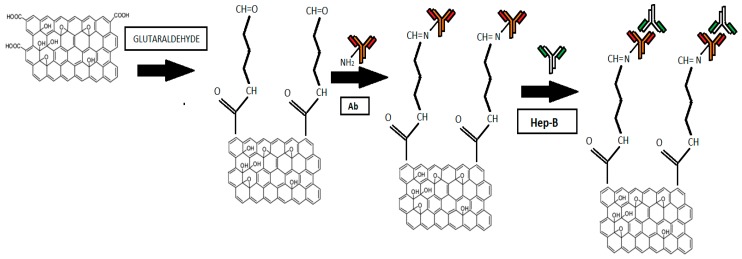
Antigen binding with grapheme.

For immobilization of anti Hep-B monoclonal antibody, (procured from Sigma Aldrich, St. Louis, MO, USA) the graphene nanogrid structure has been treated with 25%, 10% and 2.5% glutaraldehyde aqueous solutions (procured from Sigma Aldrich) for 2 h, 4 h and 24 h. After treatment, each sample has been rinsed with de-ionized water and for every parameter, optical density (O.D.) measurement has been carried out to estimate the covalent binding of graphene with glutaraldehyde. Then the graphene structure has been incubated with anti Hep-B antibody for 1 h followed by washing with phosphate buffer saline (PBS). This process activates the negatively charged carboxylic groups of graphene and improves the covalent binding of antibodies. After antibody immobilization the nanogrid structure has been incubated with different concentration of Hep-B solution for 10 min. Hep-B solution has been prepared in the range of 50 aM to 10 pM by standard process of serial dilutions [28]. The binding of antigen on graphene has been schematically represented in Figure 1.

### 2.3. FET Measurement

Electrical measurements of graphene nanogrid FETs have been performed using a Keithley 6487 instrument (A Tektronix Company, Cleveland, OH, USA) at room temperature. To facilitate electrical measurement, two ends of interdigited electrodes have been used as drain and source. A polydimethylsiloxane (PDMS) container has been glued to the surface in the region between the source and drain to avoid fluctuations due to positioning of the solution droplet. Gate has been realized using a separate platinum electrode which has been inserted into the solution in the PDMS container, through which variable gate to source voltage (*V_GS_*) has been applied. The sensing signals of the device have been recorded by monitoring the change in the drain current (*I_DS_*) for a given drain-source voltage (*V_DS_*) and *V_GS_*, when the device has been exposed to different concentrations of Hep-B solution. Further, Hep-C has been used as the non-specific antigen and complex mixtures of Hep-B and Hep-C has been prepared in buffer for testing the selectivity of the sensors. The buffer used for *I_DS_* measurement is 20 mM PBS. All the measurements have been carried out on five sets of sensors and the mean and standard deviation have been plotted.

### 2.4. Power Spectral Density Measurement

The fluctuations of *I_DS_* with time for a constant *V_DS_* and *V_GS_* have been monitored for power spectral density measurement by interfacing the Keithley 6487 unit with a PC through a serial port. The power spectral density (*S*(*f*)) of the transient fluctuations has been computed in MATLAB using Equations (1)–(3):

(1) $$\Phi \left(0\right)=\Phi \;\left(f_{0}\right)\;=\frac{1}{N^{2}}{\left|C_{0}\right|}^{2}$$

(2) $$\Phi \;\left(f_{k}\right)=\frac{1}{N^{2}}\left({\left|C_{k}\right|}^{2}+|C_{N-k}{|}^{2}\right),\;k\;=1,\;2,\;\ldots ,\;\left(\frac{N}{2}-1\right)$$

(3) $$\Phi \;\left(f_{c}\right)\;=\;\Phi \;\left(f_{\frac{N}{2}}\right)\;=\;\frac{1}{N^{2\;}}|C_{\frac{N}{2}}{|}^{2}$$

where, $f_{k}=\;\frac{k}{N\Delta t}=\;2f_{c}k/N$ (*k* = 0, 1, …, *N*/2), $\;C_{k}\;=\overset{N-1}{\underset{j=0}{\sum }}c_{j}e\;\exp\left[\frac{2\pi ijk}{N}\right]$ and Δ*t* = Sampling interval. Finally according to Parseval’s theorem, *Φ*(*f*) has been multiplied by the sampling interval Δ*t* and *N* to obtain power spectral density function, *S*(*f*). Data with equidistant time scale has been recorded and subdivided into sets, containing multiples of 2^n^ data points. For *S*(*f*) calculation, spectra consisting of 800 Fast Fourier Transform (FFT) points in frequency have been averaged for 200 s.

### 2.5. Optical Density and Raman Measurement

Optical density (O.D.) measurements have been performed in a UV-VIS spectrophotometer (Lambda 25, Perkin Elmer, Waltham, MA, USA) to estimate the number of HIgG antibodies attached to the glutaraldehyde treated graphene nanogrid structure. For estimating the binding density of HIgG antibody, HRP conjugated HIgG has been used along with a standard coloring reagent solution. The preparation details of the coloring reagent have been reported elsewhere [29]. The optical density measurements have been carried out at a wavelength of 492 nm. The O.D. value has been initially standardized with the color obtained from a known antibody solution, after reaction with the reagent. Based on this data, the number of bound antibodies on a graphene nanogrid substrate has been estimated.

For non-destructive examination of number of layers of graphene, Raman spectroscopy has been performed with a laser excitation wavelength of 532 nm and 50× objective using a LabRAM HR Evolution device (Horiba, Edison, NJ, USA). The laser power at sample has been maintained below 0.1 mW to avoid laser induced heating and sample damage. The spectrum has been accumulated over 10 s exposure for two sets. The intensity of G band has been used to determine the number of layers of graphene.

## 3. Results and Discussions

### 3.1. Graphene Characterization and Glutaraldehyde Optimization

**Figure 2:**
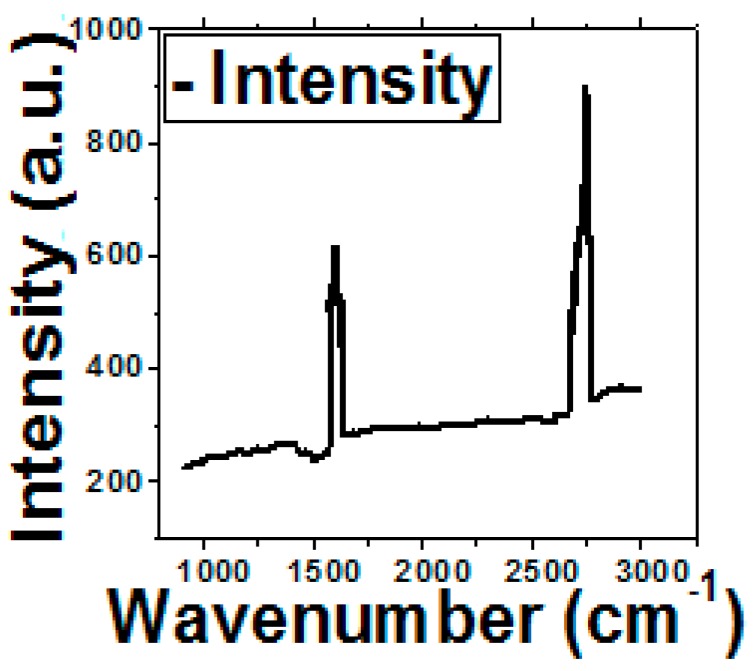
Raman Spectroscopy of graphene nanogrids.

**Figure 3:**
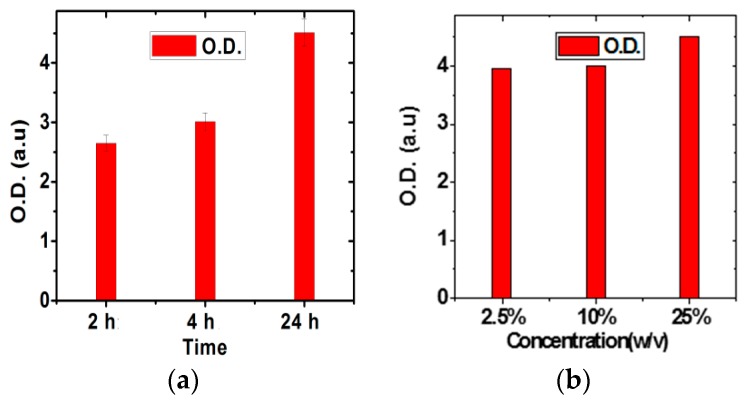
O.D. measurement for estimating antibody binding: (**a**) with different times for 25% Glutaraldehyde; (**b**) with different concentrations for 24 h treatment time.

The Raman spectrum of RGO shown in Figure 2 includes a G band at ~1585 cm^−1^ and a symmetric 2D band at ~2680 cm^−1^ with an intensity ratio of around 1.4. This confirms the presence of bilayer thickness. The antibody binding has been optimized by tailoring the glutaraldehyde concentration and treatment time for a constant incubation temperature. The concentration of glutaraldehyde solution has been selected as 2.5%, 10% and 25% for three different treatment times (2 h, 4 h and 24 h). It can be observed from Figure 3a that after 24 h treatment time, O.D. increases by almost 1.8 times compared to 2 h treatment time. This may be attributed to the fact that the orientation of the glutaraldehyde molecule is better after 24 h treatment which yields greater number of reactive CH=O groups for covalent binding with the NH_2_ groups of antibodies. The O.D. also tends to saturate after 24 h, probably because the maximum possible binding has already taken place. However, it is observed from Figure 3b that there is not much change in O.D. with variation in glutaraldehyde concentration. It is only slightly more for 25%.

### 3.2. Estimation of Sensitivity and Noise

**Figure 4:**
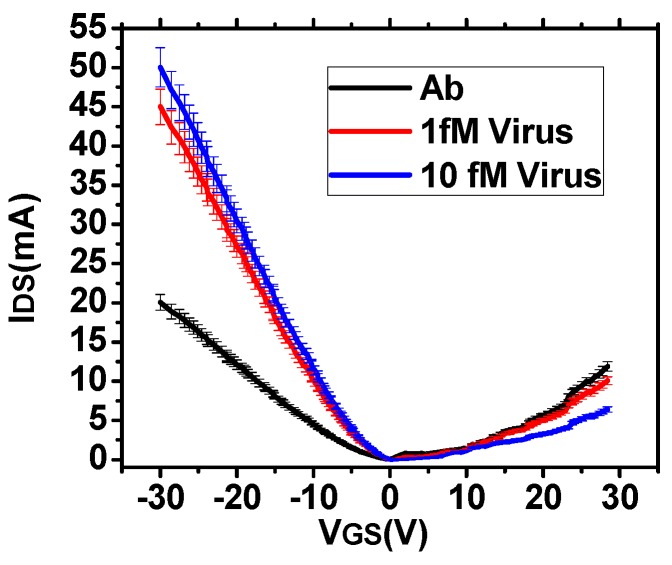
Variation of *I_DS_* with *V_GS_* after antibody binding and with increasing concentration of Hep-B virus.

*I_DS_* has been measured in presence of PBS for different *V_GS_* as shown in Figure 4 (for 25% glutaraldehyde and 24 h incubation time)for a *V_DS_* of 1 V. It has been observed that for negative *V_GS_*, the resistance is lower than that for positive *V_GS_* which indicates that the graphene layer is p-type. The glutaraldehyde treated RGO surface is negatively charged and the antibody molecules are positively charged. Hep-B virus being negatively charged [30], *I_DS_* increases with increasing concentration of the antigen for negative *V_GS_*. Further, due to increase in the carrier concentration, Dirac point shift towards right. For positive *V_GS_*, changes in *I_DS_* with Hep-B concentration are insignificant which may be attributed to the poor electrophoretic capture on the surface.

**Figure 5:**
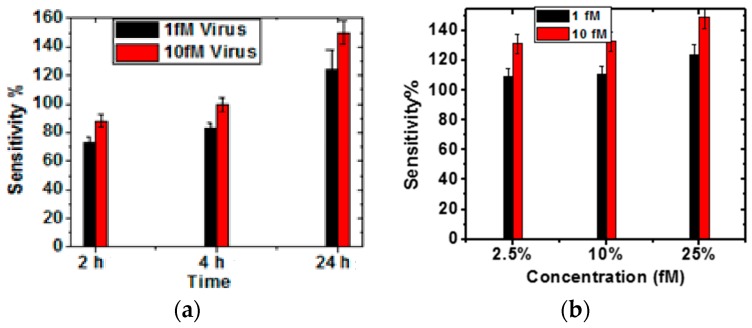
Variation of Sensitivity for different Hep-B concentrations: (**a**) with varying times for 25% glutaraldehyde; (**b**) with different concentrations for 24 h treatment time.

The sensitivity has been computed as the maximum fractional change in I_DS_ after Hep-B capture. It is observed from Figure 4, that the sensitivity is maximum corresponding to a *V_GS_* of −30 V. Thus, the variation of sensitivity with different incubation parameters of glutaraldehyde is shown in Figure 5 for *V_GS_* of −30 V. It is observed from Figure 5a,b that the sensitivity is maximum for 25% glutaraldehyde after 24 h incubation time for both the molar concentration of the analyte. This trend follows the maximum antibody binding on the sensor surface. This may be attributed to the increased capture of the Hep-B molecules and hence increased alteration in the local surface potential leading to a greater change in conductance. However, it is not sufficient to estimate only the magnitude of sensitivity since for ultra-low concentration of analyte, the stability of the *I_DS_* readings is a vital parameter for improving the sensing performance. Moreover, it is observed from Figure 5a, that with increasing sensitivity, the standard deviation also increases. Thus, noise power spectral density has been computed for all the parameters.

**Figure 6:**
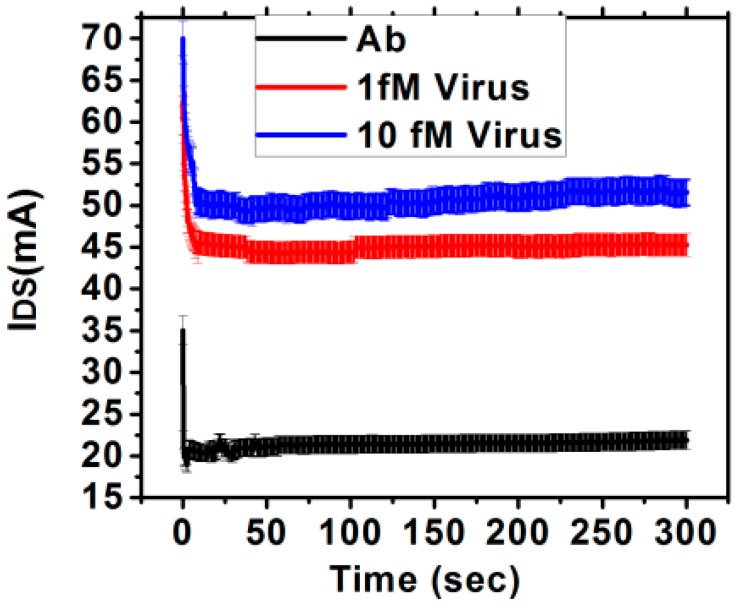
Variation of *I_DS_* with time after antibody binding and with increasing concentration of Hep-B virus.

**Figure 7:**
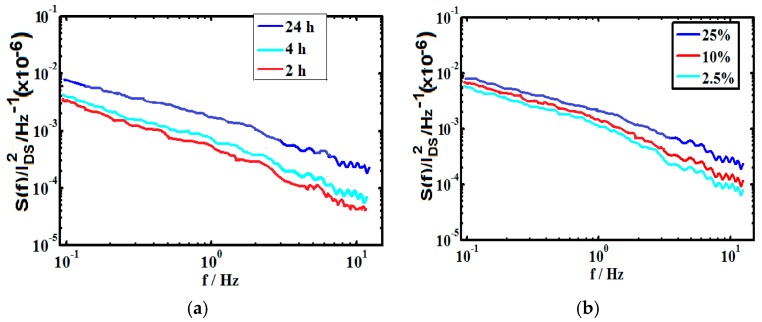
*S*(*f*)/*I_DS_*^2^ for 1 fM Hep-B corresponding to (**a**) different times with 25% glutaraldehyde; (**b**) different concentrations after 24 h.

It has been reported that the low frequency noise in electrical resistance of bilayer graphene is strongly connected to its band structure and carrier density and that the noise increases with increasing carrier density [23]. This has been attributed to two crucial reasons: interlayer charge distribution in the bilayer graphene and the nature of single particle density of states as well as the chemical potential due to the sensitivity of noise to the underlying screening mechanisms. Thus, glutaraldehyde treatment parameters which result in maximum antigen binding and sensitivity is expected to also increase the low frequency noise. The nature of the fluctuating currents for typical virus concentration is plotted in Figure 6, corresponding to 25% glutaraldehyde and 24 h incubation time. Normalized power spectrum current noise (*S*(*f*)*/I_DS_*^2^) is shown in Figure 7 for the different glutaraldehyde treatment parameters. It is observed that both sensitivity and *S*(*f*)*/I_DS_*^2^ increases with incubation time and concentration. Thus, SNR has been estimated as the ratio of sensitivity to *S/I_DS_*^2^ and shown in Table 1 for two different Hep-B virus concentration. It is observed from Table 1 that with increasing concentrations of glutaraldehyde, SNR also increases. But with incubation time, after certain hours, the SNR decreases which may be attributed to the fact that sensitivity and noise PSD change at different rates. The fractional increase of sensitivity for a change in treatment time from 2 h to 4 h is 0.468 and from 4 h to 24 h is 0.498, whereas the fractional increase in normalized PSD is 0.011 and 1.12 respectively for 1 fM concentration. The rapid increase in noise with prolonged exposure to buffer solution may originate from mobility degradation and increase of the contact resistances [11,31]. Hence, even if the change in sensitivity and noise PSD is monotonous, it is not obvious that the SNR will also follow similar trend.

**Figure 8:**
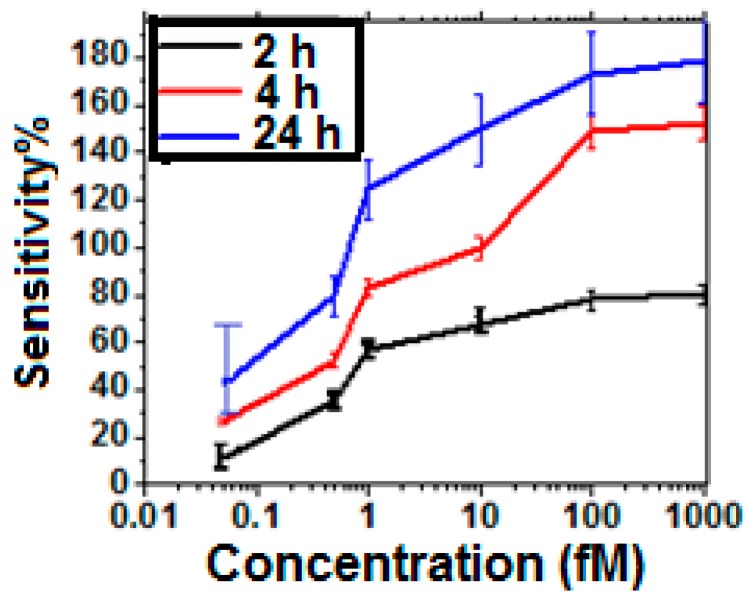
Variation of sensitivity with increasing concentration of Hep-B virus for different incubation times and 25% glutaraldehyde concentration.

**Figure 9:**
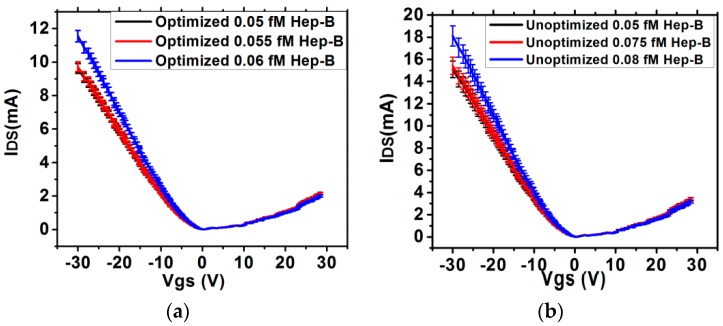
*I_DS_*-*V_GS_* characteristics for low concentration of Hep-B in (**a**) optimized and (**b**) un-optimized (24 h treated) sensor.

**Figure 10:**
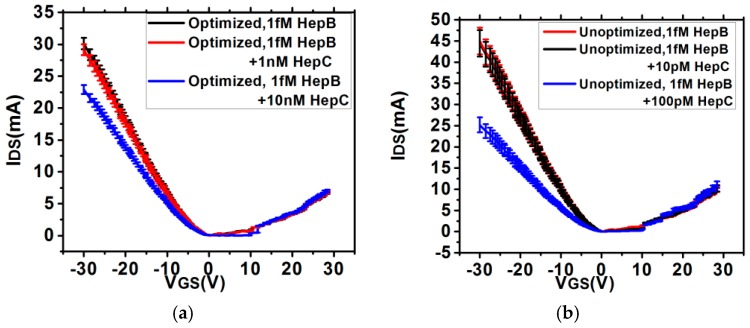
*I_DS_*-*V_GS_* characteristics for Hep-B and Hep-C mixture in (**a**) optimized and (**b**) un-optimized (24 h treated) sensor.

The impact of SNR on the sensitivity characteristics is shown in Figure 8. It is observed from Figure 8 that for a low concentration of 0.05 fM, the sensitivity magnitude corresponding to 24 h is 33% more than that for 4 h, but the deviation is significantly higher which makes the sensor unreliable. Additionally, higher SNR and lower deviation also affects the quantification accuracy. Figure 9 shows the *I_DS_-V_GS_* characteristics in presence of low Hep-B concentration for the optimized and unoptimized sensors. It is observed that the optimized sensor can quantify 0.05 fM antigen within a range of 0.05–0.055 fM which is an error of 10%. On the other hand, the unoptimized sensor detects it within a range of 0.05–0.075 fM which is an error of 50%. The impact of SNR enhancement on selectivity has also been estimated by exposing the optimized and un-optimized sensors to different complex mixtures. From the *I_DS_*-*V_GS_* characteristics shown in Figure 10, it is observed that in the optimized sensors, the *I_DS_* curve for 1 fM Hep-B is significantly reproducible even in the presence of 1 nM Hep-C, whereas in the unoptimized sensor, it shifts significantly if the Hep-C concentration exceeds 10 pM. This indicates that high SNR improves the selectivity by two orders of magnitude. This may be attributed to the increased noise in the unoptimized sensor which probably causes a significant drift in *I_DS_* due to non-specific adsorption. Hence the SNR enhancement has led to the realization of a reliable and ultrasensitive graphene nanogrid sensor with a five times and 100 times improvement in quantification accuracy and selectivity respectively.

## 4. Conclusions

This paper makes the first attempt to enhance the SNR in graphene nanosensors operating in FET mode by controlling the functionalization protocol. The proposed work is extremely relevant, especially for bilayer graphene nanostructures since the low frequency noise increases with increasing carrier density. The incubation time and the glutaraldehyde concentration have been optimized in graphene nanogrid FET sensor for maximizing the SNR. It has been observed that an incubation time of 4 h with 25% glutaraldehyde concentration results in low level detection of Hep-B virus down to 0.05 fM concentration with an appreciable sensitivity of around 30% and a standard deviation within 3%. Further, the optimized sensor has resulted in improvement of quantification accuracy by five times and selectivity by two orders of magnitude. Hence the proposed methodology for SNR optimization can be extended to different configuration of nanostructured graphene biosensors.

## Figures and Tables

**Table 1:** SNR estimation for 1 fM and 10 fM virus concentrations.

Incubation Time (h)	Concentrations of Glutaraldehyde	SNR (×10^6^) for 1 fM Hep-B	SNR (×10^6^) for 10 fM Hep-B
24	25%	327.3684	6279.0336
	10%	312.0282	6189.3021
	2.5%	311.0994	6176.0563
4	25%	461.1333	7389.6296
	10%	448.3117	7312.2811
	2.5%	446.3157	7261.6071
2	25%	317.8089	5259.6899
	10%	310.6622	5227.6785
	2.5%	303.9864	5168.1818
